# The “Sunday Blues”: a qualitative exploration of manifestations, contributing factors, and coping strategies among employees

**DOI:** 10.3389/fpsyg.2026.1882556

**Published:** 2026-09-04

**Authors:** Zhuochao Peng, Harshita Sethi, Haian Xue, Laurens A. G. Kolks, Jun Hu, Pieter M. A. Desmet

**Affiliations:** 1 Faculty of Industrial Design Engineering, Delft University of Technology, Delft, Netherlands; 2 Eindhoven School of Education, Eindhoven University of Technology, Eindhoven, Netherlands; 3 College of Design and Innovation, Tongji University, Shanghai, China; 4 Department of Industrial Design, Eindhoven University of Technology, Eindhoven, Netherlands

**Keywords:** employee wellbeing, Sunday Blues, weekend recovery, work anticipation, work-life balance, work-related stress

## Abstract

**Introduction:**

Many employees experience a decline in mood as the weekend ends, often referred to in popular culture as the “Sunday Blues.” Despite its widespread recognition, the phenomenon remains scientifically underexplored and conceptually unclear. This study examined how the “Sunday Blues” manifests, which individual and contextual factors shape the experience, and how employees cope with it.

**Methods:**

We conducted six focus groups with 26 employees in the Netherlands who frequently experienced, or had previously experienced, low moods in anticipation of the workweek. Before the focus groups, participants completed sensitization diary activities reflecting on their Sunday experiences and moods. The data were analyzed using template analysis.

**Results:**

The findings revealed that the “Sunday Blues” is a complex and multifaceted phenomenon characterized by emotional, physical, cognitive, and behavioral manifestations, including anxiety, stress, fatigue, rumination, procrastination, and social withdrawal. Three categories of influencing factors emerged: triggers, such as unfulfilled weekend expectations and anticipated challenges at work; aggravators, such as work-life imbalance and job instability; and mitigators, such as autonomy and enthusiasm for ongoing work. Participants also described a wide range of coping strategies, including self-reward, intentional weekend planning, reducing upcoming demands, and resilience-building practices.

**Discussion:**

The study clarifies the “Sunday Blues” as a multidimensional weekend-to-workweek transition experience that may be oriented both retrospectively toward the ending weekend and prospectively toward the approaching workweek. By organizing its manifestations, contributing factors, and coping strategies, the findings provide a conceptual foundation for future empirical research and intervention development.

## 1 Introduction

Imagine it is Sunday evening. The weekend seems to have passed too quickly, leaving you with a sense of sadness or disappointment. At the same time, your attention begins to shift toward the coming workweek: looming deadlines, a packed schedule of meetings, and unfinished tasks carried over from the previous week. If this scenario sounds familiar, you may have experienced the so-called “Sunday Blues.”

The term “Sunday Blues,” often used interchangeably with “Sunday Scaries” and “Sunday Night Blues,” broadly refers to the dip in mood that occurs at the end of the weekend as the new workweek approaches. A recent survey suggests this phenomenon is widespread among employees, with 80% of respondents reporting frequent experiences of it (Heitmann, 2018). Typically characterized by stress or anxiety (Tufvesson, 2022; Zuzanek, 2014), the “Sunday Blues” is associated with declines in subjective wellbeing on Sundays (e.g., Akay and Martinsson, 2009; Csikszentmihalyi and Hunter, 2003; Mihalcea and Liu, 2006; Zuzanek and Mannell, 1993). When experienced repeatedly, the stress and negative affect associated with this transition period may also have implications for health (e.g., Cohen et al., 2015; Kessler, 1997).

The “Sunday Blues” has consequently received considerable attention in popular culture. Sunday-night moods are frequently expressed through memes on social media, while blogs, podcasts, and wellness platforms regularly discuss the phenomenon and recommend ways of managing it (e.g., Headspace, 2021; Pinsker, 2020). However, scientific understanding has not kept pace with this cultural recognition. Existing research offers indications of a Sunday decline in mood, but provides limited insight into the phenomenon as it is lived and understood by employees. In particular, it remains unclear how the “Sunday Blues” manifests beyond general labels such as low mood, what factors or conditions shape it, and how people respond when it occurs.

To address these gaps, the present study qualitatively explores employees’ experiences of the “Sunday Blues,” focusing on its manifestations, contributing factors, and coping strategies. The following literature review situates the phenomenon at the intersection of three bodies of research: weekly affective rhythms, recovery during the weekend, and anticipation of work during nonwork time. Together, these perspectives clarify what is already known about the weekend-to-workweek transition and what remains to be understood about the “Sunday Blues” as a concrete lived experience.

## 2 Literature review

### 2.1 Weekly affective rhythms and the “Sunday Blues”

The “Sunday Blues” can first be situated within research on weekly affective rhythms. The seven-day week is not merely a chronological unit; it is also a socially organized rhythm that structures work, leisure, rest, and social expectations (Zerubavel, 1989). Psychological research has therefore examined whether affective experiences vary systematically across the weekly calendar. Early work by Larsen and Kasimatis (1990), for instance, identified a weekly rhythm in mood while also revealing individual differences in the extent to which people’s moods follow this pattern. Subsequent research has investigated day-of-week effects in more detail, often through culturally recognizable ideas such as “Blue Monday,” “Hump Day” (Wednesday), “Thank God It’s Friday” (TGIF), and the weekend effect. Overall, the findings suggest that weekly mood patterns exist, but are more complex than those labels imply. For example, Areni (2008) showed that reported weekly moods can be shaped by expectations about work and discretionary time, while large-scale studies have found more positive mood and higher wellbeing on weekends and Fridays but inconsistent evidence for a distinct “Blue Monday” effect (Helliwell and Wang, 2014; Stone et al., 2012). These patterns also vary across societies and cultural contexts (Tsai, 2019).

Sunday occupies an interesting position within this weekly rhythm. As part of the weekend, it belongs to a period generally associated with enhanced mood, vitality, and wellbeing compared with weekdays or work periods (e.g., Helliwell and Wang, 2014; Ryan et al., 2010; Stone et al., 2012). At the same time, Sunday marks the point at which the weekend begins to give way to the workweek. Studies have documented declines in mood or wellbeing as Monday approaches (e.g., Akay and Martinsson, 2009; Csikszentmihalyi and Hunter, 2003; Mihalcea and Liu, 2006; Zuzanek and Mannell, 1993), pointing to the experience popularly described as the “Sunday Blues.”

Despite these indications, the “Sunday Blues” remains conceptually underdeveloped. Terms such as “Sunday neurosis” and “Sunday night blues” have been used to describe negative affect near the end of the weekend, but their scope and meaning are not clearly established (e.g., Areni and Burger, 2008; Suhara et al., 2017). Existing accounts variously describe “emotional discomfort at the doorstep of a new week” (Zuzanek, 2014, p. 7), “anxiety or dread experienced the day before heading back to work after the weekend” (Tufvesson, 2022, p. 50), and feeling “depressed by virtue of heralding in another long week” (Mihalcea and Liu, 2006, p. 142). While these descriptions locate the experience temporally and establish its negative affective tone, they do not clarify whether the “Sunday Blues” is a unitary mood state or a more multifaceted experience. Nor do they explain what employees attend to, think about, or do when it emerges. To deepen understanding of the phenomenon, it is useful to consider the psychological processes associated with both sides of the transition: recovery during the weekend and anticipation of the workweek ahead.

### 2.2 Recovery during the weekend

Recovery research starts from the premise that work demands consume psychological and physiological resources that must be restored during nonwork time (Meijman and Mulder, 1998; Sonnentag, 2001). Weekends are particularly important in this process because they provide an extended period away from work in which employees can replenish depleted resources and sustain wellbeing (Fritz et al., 2010; Fritz and Sonnentag, 2005).

However, weekends are not inherently restorative. Social, physical, and creative activities during the weekend tend to facilitate recovery, whereas work-related activities and nonwork hassles can impede it (Fritz and Sonnentag, 2005; Ginoux et al., 2021). Recovery is also supported by experiences of psychological detachment, relaxation, mastery, and control (Binnewies et al., 2010; Sonnentag and Fritz, 2007). Such experiences predict more positive affect at the end of the weekend, with some benefits extending into the following workweek (Fritz et al., 2010). Psychological detachment has received particular attention in this regard. Continuing to think about work during the weekend can interfere with recovery, for example by impairing sleep (Syrek et al., 2017), while persistent difficulty detaching from work has been associated with greater strain and poorer wellbeing (Sonnentag and Fritz, 2015).

This literature suggests that employees may arrive at Sunday evening with very different levels of restored resources, depending on what occurred during the weekend and how they experienced it. Employees who perceive their weekend as insufficient, disrupted, or unfulfilling may therefore experience its ending more negatively. However, while recovery research primarily explains how the preceding workweek and the quality of nonwork time carry forward into Sunday, it says less about how thoughts of the approaching workweek begin to shape experience before the weekend has ended. For that, research on work anticipation offers a complementary perspective.

### 2.3 Work anticipation during nonwork time

Occupational health research suggests that employees may think about future tasks, workloads, or responsibilities before a work period begins, particularly because much work is scheduled in advance and is therefore preceded by anticipation (Casper and Sonnentag, 2020; Mohammed and Nadkarni, 2011). Such thoughts may give rise to anticipatory work-related affect when upcoming work demands are appraised as threatening, uncertain, or difficult to manage.

Two relevant forms of anticipatory affect are stress and anxiety. Anticipatory stress refers to stress-related responses that arise before a demanding event actually occurs (Meurs and Perrewé, 2011; Monat et al., 1972). Anticipatory anxiety, by comparison, refers to anxiety experienced in relation to uncertainty about a possible future threat (Grillon, 2008; Grupe and Nitschke, 2013). Although anticipatory stress foregrounds the appraisal of future demands and anticipatory anxiety foregrounds uncertainty and potential threat, both demonstrate that future events can already shape present affective experience.

These anticipatory processes may be particularly prominent on Sunday. Although Sunday remains part of nonwork time, the coming workweek may already become psychologically present. Previous research suggests that the benefits of weekend recovery can begin to fade on Sunday evening, possibly as employees anticipate the following week’s workload (Casper and Sonnentag, 2020; Hülsheger et al., 2014; Rook and Zijlstra, 2006). The boundary between weekend and workweek is therefore not only chronological; it may also be crossed psychologically before work formally resumes.

Taken together, the three bodies of literature position the “Sunday Blues” as an affective experience embedded in a socially structured weekly transition. Weekly rhythm research establishes that mood can change as Monday approaches; recovery research suggests that the experience may depend partly on how restorative the weekend has been; and work anticipation research explains how future work demands can intrude upon present nonwork time. This synthesis points to a potentially two-directional experience, oriented retrospectively toward the weekend that is ending and prospectively toward the workweek that is approaching. However, existing research has examined these processes separately and has not yet clarified how they come together in employees’ lived experiences, which factors or conditions influence the transition, or how employees attempt to manage it.

## 3 Present study

The limited understanding of the “Sunday Blues” constrains its conceptual development and provides an insufficient empirical basis for identifying how the experience might be alleviated or prevented. The present study therefore qualitatively explores how employees experience and cope with the “Sunday Blues.” Specifically, it addresses the following research questions:

- How does the “Sunday Blues” manifest in employees’ lived experiences?
- What individual and contextual factors shape it?
- How do employees cope with it?

## 4 Methods

We conducted focus groups because they are well suited to generating rich accounts of socially recognizable experiences through interaction and collective reflection (Acocella, 2012). This section describes participant recruitment, the study procedures, data analysis, and researcher reflexivity.

### 4.1 Participants

We used convenience sampling to recruit participants from a university-based research pool and our professional networks in the Netherlands. To participate, individuals were required to: (1) be at least 18 years old; (2) be employed; (3) typically begin their workweek on Monday; (4) frequently experience, or have previously experienced, low moods on Sundays in anticipation of the new workweek; (5) be willing to attend an in-person session at the university; and (6) feel comfortable conversing in English.

The final sample comprised 26 participants across six focus groups, with three to five participants in each group (see Table 1). The relatively small groups were intended to support in-depth discussion while allowing each participant sufficient opportunity to contribute (Clarke and Braun, 2013). Each participant received a fifty-euro voucher as compensation. Ethical approval of this study was obtained from the Human Research Ethics Committee of Delft University of Technology (TU Delft, the Netherlands), and all participants provided informed consent before taking part.

**Table 1:** Overview of participants.

Group name	Group size	Participant code	Age	Working domain
Group 1	4	G1P1, G1P2, G1P3, G1P4	52–65	Healthcare, transportation and logistics, food and beverage, elementary education
Group 2	3	G2P1, G2P2, G2P3	42–64	Standardization and certification, professional services, healthcare
Group 3	5	G3P1, G3P2, G3P3, G3P4, G3P5	29–65	Wholesale and retail sales, professional services, semi-government, higher education, information technology and services
Group 4	4	G4P1, G4P2, G4P3, G4P4	26–32	Higher education, information technology and services
Group 5	5	G5P1, G5P2, G5P3, G5P4, G5P5	26–29	Higher education
Group 6	5	G6P1, G6P2, G6P3, G6P4, G6P5	25–29	Higher education, information technology and services

### 4.2 Procedure and research materials

#### 4.2.1 Sensitization

Before the focus groups, participants completed a sensitization task designed to heighten their awareness of their Sunday contexts, routines, and mood shifts and to support concrete reflection during the group discussions. This task involved two online diary entries completed before bed on two consecutive Sunday nights and was organized into four sections.

First, participants reflected on the highlights and low points of their weekend. Second, they described their mood both during Sunday daytime and at the time of writing by selecting characters from Pick-A-Mood (Desmet et al., 2016) and explaining their choices. Third, they noted their expectations and apprehensions about the upcoming week, highlighting specific events they anticipated or dreaded. Lastly, they reflected on their general awareness of everyday moods and their strategies for managing low moods on Sunday evenings.

As a final preparation step, participants revisited their two diary entries 1 day before the focus group. Further details about the Sunday night diary are provided in Supplementary material.

#### 4.2.2 Focus group

Each focus group followed five stages: (1) warm-up, (2) exploring the “Sunday Blues,” (3) understanding influencing factors, (4) identifying coping strategies, and (5) wrap-up (see Supplementary material for the full guide).

We began the warm-up with an icebreaker in which participants shared their current moods. We then addressed ethical considerations, such as data privacy, and encouraged participants to engage in dialogue with one another rather than respond solely to the researcher.

In the second stage, we introduced our working definition of the “Sunday Blues” as “the not-so-great feeling we get when the weekend is ending and the workweek is just around the corner.” We first asked participants to discuss their general experience, frequency, and when they typically notice the “Sunday Blues” setting in. We then inquired about specific aspects of their experience, including their mental and physical feelings, changes in perceptions of their own lives and surroundings, their reactions to others, and any behaviors and preferences that arise.

The third stage focused on the reasons behind the “Sunday Blues.” Initially, we allowed for open discussion, encouraging participants to reflect on and share personal experiences. To prompt further insights, we provided a set of “factor cards” (see Supplementary material) that illustrate potential influencing factors, which were identified from diaries participants had completed in the prior sensitization task. We asked participants to identify which of these factors resonated with their experiences beyond what had already been discussed in the focus group.

In the fourth stage, participants discussed coping strategies. They first shared their typical approaches, followed by a review of “strategy cards” (see Supplementary material) derived from sensitization diaries to stimulate further ideas.

Finally, in the wrap-up, we asked participants to describe how they would ideally like to feel on a Sunday evening as an alternative to experiencing the “Sunday Blues.” To support coping beyond the focus group session, they received a self-care booklet offering recommended strategies and activities (see Supplementary material).

All focus groups were moderated by the first author, with notes taken by the second author. Each session, lasting between 2 and 3 h, was audio-recorded for later analysis. Data collection took place over the course of 1 month.

### 4.3 Data analysis

All audio recordings were transcribed verbatim, with participant names, organization names, and geographical locations anonymized to protect privacy. We conducted template analysis, a more structured form of thematic analysis commonly used in qualitative psychology research, which combines inductive coding with the development and use of a coding framework (Brooks et al., 2015; King, 2012). Drawing on King’s guidance (2012), our analysis involved six key steps: (1) familiarization with the data; (2) coding; (3) clustering codes; (4) developing an initial coding template; (5) iteratively refining and finalizing the template; and (6) reporting the findings.

Familiarization occurred during transcription. Two researchers (the first and second authors) independently conducted open coding on two randomly selected transcripts (from a total of six) using ATLAS.ti, generating initial codes and organizing them into thematic clusters. To support the analysis of participants’ coping strategies for the “Sunday Blues,” we incorporated Desmet’s (2015) categorization of mood-regulating activities as a set of *a priori* themes; however, these were treated as provisional and open to revision or removal as the analysis progressed (King et al., 2017). The preliminary codes and themes served as the basis for a discussion between the two researchers to align perspectives and develop an initial coding template. This template was then applied to the remaining transcripts. Both researchers independently coded the data and met regularly to review findings and revise the template in response to emerging insights. This iterative process allowed for the addition, modification, or redefinition of codes and themes throughout the analysis. The final coding template was reviewed and agreed upon by all authors when reporting the findings.

### 4.4 Researcher reflexivity

The research team approached this study from backgrounds in design research, emotion psychology, and wellbeing, with a shared interest in everyday mood experiences and mood regulation. Team members were personally familiar with the “Sunday Blues” to varying degrees; the first author, who moderated all focus groups, experiences it frequently. This familiarity may have supported sensitivity to participants’ descriptions and helped recognize nuanced aspects of the phenomenon. At the same time, it may have introduced assumptions about what the “Sunday Blues” feels like, how it unfolds, and what kinds of coping strategies are meaningful. We remained attentive to these potential influences when developing the prompts, facilitating the group discussions, interpreting participants’ accounts, and iteratively refining the coding template.

## 5 Results

Through six focus group discussions, we examined how the “Sunday Blues” manifests, the factors influencing it, and the coping strategies employees use. In this section, we present our findings by organizing codes and illustrative participant statements into structured tables under each thematic area, and providing narrative accounts of them to ensure both clarity and detail.

### 5.1 Manifestations of the “Sunday Blues”

How does the “Sunday Blues” manifest in employees’ lived experiences? Our analysis revealed five thematic clusters: the general qualities of the “Sunday Blues,” along with its affective, physical, cognitive, and behavioral components.

#### 5.1.1 General qualities

Nine key characteristics of the “Sunday Blues” emerged (see Table 2). The experience is often elusive and abstract, making it difficult to pinpoint its exact nature or causes. For many, it is mild to moderate in intensity—more of a subtle emotional undercurrent than an overwhelming state. However, in certain situations, such as when anticipating a high-pressure workweek, the feeling can intensify significantly. Despite these variations, the “Sunday Blues” is generally short-lived, typically peaking on Sunday evening or night before fading as the new week begins. Its focus can range from a diffuse sense of unease and specific responses to stressors, such as looming deadlines or interpersonal challenges at work. Emotionally, it tends to be multilayered, often involving a blend of feelings such as sadness, anxiety, and regret. These layers shift in prominence depending on personal circumstances. The experience is also dynamic: it may build gradually, spiral downward, or reinforce itself through patterns of avoidance or inaction. Notably, the “Sunday Blues” is not a consistent or routine occurrence. Its presence depends on situational factors such as workload, social interactions, and ongoing life events. Each episode carries unique nuances, shaped by the week’s specific context. Furthermore, the phenomenon has a social dimension, often unfolding within close relationships or among peer groups who share Sunday evenings, where one person’s mood may influence others or shape the collective atmosphere.

**Table 2:** General qualities of the “Sunday Blues.”

Code	Explanation	Example participant statement(s)
Intangibility	The “Sunday Blues” is a vague, abstract experience, often difficult to define or attribute to a specific source.	“For me in general, the ‘Sunday Blues’ is very, very vague … I did not know what it is, why it came, and what’s the cause.” (G5P4)
Low intensity	The emotional tone of the “Sunday Blues” is typically mild or moderate, although it can occasionally be intense depending on circumstances.	“It’s not very heavy, but I know it’s there.” (G1P1) “Every week, I felt very strong ‘Sunday Blues’ because I had a meeting with my supervisor on Monday morning” (G5P3).
Limited duration	The “Sunday Blues” is transient in nature, typically confined to Sunday evening or the period leading up to Monday.	“Actually, they are very short, momentary feelings … [only] till the start of Monday morning.” (G6P2)
Diffuseness and focus	The “Sunday Blues” may manifest as a broad, undefined sense of unease, or be tied to specific stressors, such as particular people or events.	“I just had a slight nag, like something pulling at you. This is [more] general than others because it could [also] be something specific, and then it’s different. Then you might be saying, ‘Oh, the idiot you are into, or the team and the people you have to manage, or something like that.’” (G2P2)
Multilayered composition	Rarely singular in nature, the “Sunday Blues” often involves a combination of multiple, co-occurring emotional states.	“Basically, my ‘Sunday Blues’ was like you are tired, distracted, postponing, a bit of bored … Also, that anxious feeling was there.” (G6P5)
Dynamic progress	The “Sunday Blues” may build gradually or evolve over time, sometimes reinforcing itself through avoidance or inaction.	“As the time is approaching, the negative emotion is getting more.” (G6P4) “I’m like in a downward spiral, sucking everything [negative] with me.” (G1P3) “One of the things I would ideally want to do is to call my family. But if I feel bad at the ‘Sunday Blues,’ and then I feel like, ‘Oh, now I really do not feel like calling them.’ And then I regret that decision.” (G4P1)
Irregular occurrence	The “Sunday Blues” does not occur every week; its occurrence is shaped by situational factors like workload, interpersonal dynamics, and broader life events.	“It depends on how much pressure is there on the work deadline, how relaxed are the others working with you, or if there’re other things going on.” (G2P2)
Nuances with each occurrence	Each experience of the “Sunday Blues” can feel slightly different, influenced by the context of that particular week.	“There are different kinds of experiences, but the overwhelmed feeling of not being able to do all the things I want to do, that’s relatively a recurring feeling … If I’m frustrated with someone, like, I have an issue with a colleague, that would be a different kind.” (G4P1)
Social contagion	The “Sunday Blues” can be socially contagious, spreading within relationships and affecting group dynamics.	“It’s my husband [and I] not having the same feeling of satisfaction about the weekend … then you get arguing. Actually, it’s his ‘Sunday Blues,’ but it gets spreading to me.” (G1P4) “When you say goodbye to your friends on Sunday evening, if someone is complaining, ‘Oh no, the next day is Monday, we have to work,’ I feel everyone is not that happy. It also affects [me].” (G4P4)

#### 5.1.2 Affective components

Participants reported a range of emotional experiences associated with the “Sunday Blues” (see Table 3). At the front was anxiety, often linked to worries about the upcoming week. These included fears of potential difficulties in ongoing projects, underperformance, or receiving negative feedback. Stress was also frequently mentioned, though it had a different focus. While anxiety centered on uncertainties, stress was more about feeling overwhelmed by known responsibilities, such as unfinished tasks from the previous week or approaching deadlines. Sadness or melancholy was another common experience, reflecting a sense of loss as the weekend came to an end. Many participants expressed regret over not meeting personal goals or making full use of their weekend, often accompanied by self-blame. In contrast, some described a sense of emptiness, arising from the abrupt shift from a fulfilling weekend to a quieter, less stimulating Sunday evening. This quiet period also appeared to bring on feelings of helplessness or inadequacy, particularly when participants reflected on broader challenges or uncertainties that felt beyond their control.

**Table 3:** Affective components of the “Sunday Blues.”

Code	Explanation	Example participant statement(s)
Anxiety	Feeling uneasy or apprehensive about uncertainties in the upcoming week, often fearing negative outcomes.	“Basically just being anxious about the next day in general because I do not know what the next day will bring in.” (G3P2) “I was a bit nervous about how the workshop would go and how the patient would react.” (G6P5)
Stress	Feeling overwhelmed or pressured by upcoming responsibilities, workload, or expectations for the week ahead.	“Sometimes I feel like there’s just too many things and no way of doing them all. So, then, I get overwhelmed.” (G4P1) “Normally, I have a clear goal of what is going to be achieved in the next week. So, on Sunday night, these goals will immediately go to my mind and make me feel stressful.” (G6P2)
Sadness	Feeling down or melancholic as the weekend, a time for rest and enjoyment, is over.	“When it’s turning dark [on Sunday], I feel a bit emotional.” (G6P4) “What I meant by ‘sad’ is more about why the weekend is too short.” (G6P2)
Regret	Feeling remorseful for not making better use of the weekend or failing to achieve personal goals.	“I’ve got regret for things that I did want to do on the weekend [but] did not do.” (G3P2) “The regret would be another situation where I feel like I’ve wasted time on something that did not turn out as good as I thought.” (G4P1)
Emptiness	Feeling a sense of void or lack of purpose as the weekend winds down and activities taper off.	“All of a sudden, it’s gone. It’s over. And then your schedule is empty. But you also feel empty on the inside because you have nothing to live for or to look forward to.” (G1P3)
Helplessness	Feeling powerless or unable to manage one’s emotional states or anticipated challenges in the week ahead.	“It’s about my uselessness … because at that moment, I cannot do anything about it.” (G5P4)

#### 5.1.3 Physical components

Participants described a variety of bodily sensations associated with the “Sunday Blues” (see Table 4). A common experience was fatigue, characterized by low energy and often attributed to either an overly active weekend or lingering exhaustion from the previous workweek. In contrast, some reported feeling restless—a sense of inner agitation that made it difficult to unwind or stay still. Several participants noted specific physical discomforts, such as tightness or pressure in the head that sometimes escalated into headaches, and an accelerated heart rate that heightened their sense of unease. Alongside these, many described a sensation of heaviness or tension in the stomach, while others experienced strong cravings for particular foods, especially those offering comfort or stimulation. Muscle stiffness or tension was another prevalent experience, reflecting how stress was carried in the body, and for some, this emotional strain left them feeling vulnerable and on the verge of tears.

**Table 4:** Physical components of the “Sunday Blues.”

Code	Explanation	Example participant statement(s)
Fatigue	A general sense of tiredness or low energy throughout the body, often tied to overexertion during weekend activities or residual exhaustion from a demanding workweek.	“Sometimes feeling exhausted while I was doing particularly active things during that day. So, an errand exhaustion.” (G3P5) “Maybe mentally I’m getting more relaxed during the weekend, but physically I’m getting more tired sometimes, especially if I have a really busy week.” (G4P3)
Restlessness	A feeling of agitation and inability to settle or relax, marked by a persistent but unfocused urge to take action.	“You cannot sit down, and you feel you have to do something, but you do not do anything really.” (G3P4)
Head pressure	A sensation of tightness or heaviness in the head that could develop into headaches.	“I feel there’s some kind of pressure, overpressure, in my head.” (G3P5)
Increased heart rate	A noticeable acceleration in heartbeat that could heighten awareness of physical unease.	“I can feel my heart rates are going this higher.” (G3P5)
Stomach discomfort	An uneasy, heavy, or aching feeling in the stomach, sometimes spreading through the body.	“Nearly an ache, it’s a feeling of misery inside your stomach. Something heavy on your stomach.” (G1P3)
Food cravings	Strong desires for specific or comforting foods, usually driven by emotional needs rather than hunger.	“It’s not like I feel hungry. It’s like I feel my needs for the food, especially the spicy food.” (G6P4)
Muscle tension	Felt tightness or stiffness in muscles, often associated with stress or emotional strain.	“I feel being tensed up … I feel it in my body that the tension is over my muscles.” (G3P3)
Tearfulness	A sensation of being close to tears, typically in response to anxiety or emotional overload.	“If I’m anxious and worried, I do feel close to tears.” (G3P2)

#### 5.1.4 Cognitive components

Participants described multiple thought patterns linked to the “Sunday Blues” (see Table 5). A pervasive pessimistic mindset often shaped how they interpreted their situations and life in general, with work challenges perceived as insurmountable and their own efforts deemed lacking value. Many reported a constant mental rehearsal of the upcoming week, repeatedly anticipating responsibilities and work demands, which made it difficult to stay in the present moment and relax. In contrast, others experienced difficulty concentrating or organizing their thoughts as if trapped in a mental fog, often leaving them feeling overwhelmed and disoriented. Excessive analysis was also common; participants found themselves caught in prolonged episodes of rumination, revisiting past decisions or imagining negative outcomes. Underlying many of these patterns was a strong tendency toward self-criticism, with participants engaging in harsh self-judgment and blame over perceived failures or an inability to maintain balance between work and personal life.

**Table 5:** Cognitive components of the “Sunday Blues.”

Code	Explanation	Example participant statement(s)
General negative outlook	A pervasive tendency to interpret situations, tasks, or life in general through a pessimistic lens.	“Everything seems so negative. You experience it as worse and harder than it might actually be.” (G1P3) “I think very little of myself, so then my life has no value.” (G3P2)
Preoccupation with upcoming tasks	Persistent mental focus on upcoming responsibilities and work demands, hindering the ability to stay present and relax.	“All the tasks are going to be getting in fluctuating in your head, like, ‘I need to do this, I need to present something, etcetera, etcetera.’” (G6P1)
Difficulty concentrating	A sense of mental fog or lack of clarity regarding current conditions or tasks, resulting in feelings of overwhelm or disorientation.	“I have a ‘foggy’ mind. The tasks I have to do are not clearly listed, so it feels like I have so much to do.” (G5P2)
Excessive analysis	Overanalyzing various aspects of one’s life, such as re-evaluating past decisions, second-guessing social interactions, or imagining worst-case scenarios.	“I’m spiraling about negative things … for example, why did I choose to come to [this country]? I can really go back to my decisions.” (G4P3) “I get a little bit paranoid. … I’m like, ‘Does somebody have this alternative motive for something?’” (G3P5)
Self-criticism	Harsh self-evaluation or blame, often focused on personal shortcomings or inability to manage responsibilities or life in general.	“I get a little negative about myself. I’m giving myself negative feedback.” (G1P4) “It’s me overthinking, ‘Why I’m letting my work get so busy? Why am I doing this to myself? Why am I making it so difficult?’” (G2P1)

#### 5.1.5 Behavioral components

Participants described distinct behavioral patterns accompanying their experiences of the “Sunday Blues” (see Table 6). Most prominent was a tendency toward procrastination or inaction, with many avoiding tasks by sitting or lying down for prolonged periods, as if waiting for their situations to resolve on their own. This passivity often occurred alongside a quiet social withdrawal from others; participants described turning down invitations or disengaging from conversations to protect themselves from emotional drain. Decreased patience or tolerance also surfaced, with several noting a heightened sensitivity to minor frustrations and a tendency to respond more harshly than usual. In contrast, others attempted to regain a sense of control through compulsive activities—cleaning, organizing, or engaging in busywork that seemed more about movement than purpose. Changes in sleep were another notable pattern: for some, fatigue led to unusually early bedtimes, while others struggled with insomnia or delayed sleep, preoccupied with anxious thoughts about the week ahead.

**Table 6:** Behavioral components of the “Sunday Blues.”

Code	Explanation	Example participant statement(s)
Procrastination or inaction	Avoiding any active tasks by remaining still for extended periods, passively waiting for distractions or hoping things will change on their own.	“I’m sitting, waiting … I do not know for what I’m waiting.” (G1P2) “I feel lazy … I feel like I really need a stretch, but I do not have the motivation or energy to ask myself to start stretching.” (G6P4)
Social withdrawal	Distancing oneself from social interactions, such as ignoring messages, declining invitations, or reducing engagement in conversations.	“When my neighbor calls, I do not open the door.” (G1P2) “If someone asks me for help on Sunday evening, then I might not be that willing to do it.” (G4P4)
Decreased patience or tolerance	Becoming easily annoyed by minor difficulties or inconveniences and reacting to others more negatively or abruptly than usual.	“I can be upset quicker. I wrote in my diary about how I baked some pancakes and only three were good … then I get quickly irritable.” (G2P1) “I will not be that patient … and also my tone will be not that positive … People [can] really feel that I’m kind of annoyed.” (G4P2)
Compulsive activities	Engaging in repetitive or seemingly unnecessary activities, not because of a specific purpose but due to an urge to stay busy.	“I clean something up, organize it, bring order to chaos … but then I would say, ‘Do I really have to be doing this right now?’” (G2P2)
Changes in sleep patterns	Experiencing changes in sleep, such as going to bed unusually early due to fatigue, struggling with insomnia due to racing thoughts, or staying up late to extend the weekend.	“You cannot go to sleep because you are having things in your mind, and then that fucks you up every second.” (G3P5) “I feel sleepy … so I need to go to bed earlier.” (G4P2) “It’s a feeling of just wanting to extend the weekend as longer as I can, so I stay up late.” (G6P2)

### 5.2 Contributing factors to the “Sunday Blues”

What individual and contextual factors shape the “Sunday Blues”? Three categories of influencing factors emerged during our analysis: triggers directly leading to the “Sunday Blues,” aggravators intensifying it, and mitigators alleviating its negative effects.

#### 5.2.1 Triggers

Participants reported various triggers for the “Sunday Blues” (see Table 7). As Sunday evening unfolded, many described a sudden realization of losing a time for fun and relaxation, often leaving them feeling sad. When weekends were packed with social events or household chores, participants commonly reported feeling mentally and physically depleted, which contributed to a sense of unpreparedness for the week ahead. In contrast, when the weekend failed to meet their expectations—due to unfinished tasks, disappointing experiences, or insufficient personal time—feelings of regret often emerged and lingered into the evening. Beyond reflecting on the weekend, participants also described a constant anticipation of the upcoming week, which frequently triggered anxiety, especially when they faced uncertainty around work demands, potential feedback, or social interactions. This anticipation became particularly stressful when they foresaw overloaded schedules or especially difficult responsibilities. For many, Monday itself emerged as a stressor, seen as a symbol of routine, responsibility, and, in some cases, the most demanding day of the week due to recurring meetings and heavy workloads. In addition to these psychological factors, tangible cues, such as early morning alarms or work-related messages, were frequently noted as stressful reminders of the return to work.

**Table 7:** Triggers for the “Sunday Blues.”

Code	Explanation	Example participant statement(s)
Loss of leisure	The realization that the weekend—a time of freedom, relaxation, and enjoyment—is ending.	“My weekend of free time is almost over.” (G3P3)
Insufficient rest	A lack of physical and mental recovery due to weekends filled with excessive social activities, family obligations, or accumulated chores.	“You do not rest enough because you are too busy being socially with other people.” (G5P2)
Unfulfilled weekend expectations	The belief that the weekend has not met personal expectations, whether due to missed plans, unsatisfying activities, or a lack of meaningful time for oneself.	“If there were something planned, but I did not really do that, that makes me feel a bit unsatisfied with myself.” (G4P4) “If I did not spend enough time for myself, I will feel a little bit disappointed.” (G6P2)
Uncertainty about the week ahead	A lack of clarity around what the upcoming week may involve, including unclear workloads, unforeseen challenges, or uncertain feedback that could affect progress.	“I do not know what might happen, and I do not know whether I’ll be able to handle them. And that makes me anxious.” (G5P4) “The panic moment you get on Sundays is like, ‘Oh no, they are going to give me some feedback [tomorrow]’ … because if the feedback is not in favor of you or the work you did, that’s going to be in a dilemma phase.” (G6P1)
Anticipated challenges at work	The projection of a challenging workweek, including heavy workloads, looming deadlines, unresolved issues, structural barriers in the job, or difficult social dynamics at work.	“One is the upcoming heavy workload because we get more and more work.” (G3P2) “There’s a lot of things blocking me in my work, either software-related or people-related.” (G3P5) “It’s been stressful at work with relationships and with people doing the power plays and stuff.” (G2P2)
Monday as a stressor	Monday as a starting point of hectic routines, often perceived as the busiest day of the week due to recurring meetings or early work commitments.	“The whole rush is starting up again.” (G2P2) “For all of us, it always is a busy Monday—it’s quite one day.” (G1P2)
Tangible reminders	Specific environmental or digital cues—such as emails or alarms—that interrupt the weekend mindset and signal the return to responsibilities.	“When you go to bed, if you set the alarm clock, the blues kick in.” (G2P3) “If I get working emails on Sunday night, I will be extremely anxious.” (G6P2)

#### 5.2.2 Aggravators

Participants described a range of factors that intensified their experience of the “Sunday Blues” or increased the likelihood of its onset (see Table 8). Leading among these was an overall imbalance between personal and professional life, particularly the struggle to mentally disengage from work over the weekend, which often left participants prone to mental fatigue. Starting a new job also emerged as a key aggravator: for some, it involved major life transitions, such as relocation or physical separation from loved ones; for others, the uncertainty surrounding a new role or probationary status served as a continuous source of insecurity. In parallel, a strong drive for visibility or success at work frequently introduced extra pressure, often prompting comparisons with peers and, in many cases, resulting in a sense of inadequacy or underachievement. Additionally, several participants described how ongoing personal ill-being left them in a more emotionally vulnerable state, amplifying feelings of reluctance or even dread in anticipation of the workweek.

**Table 8:** Aggravators for the “Sunday Blues.”

Code	Explanation	Example participant statement(s)
Work-life imbalance	Blurred boundaries between personal and professional life, often involving perceived obligation to work during weekends and difficulty switching off.	“I do not have a clear distinction [between] work and leisure. I’m still hopeful that there’s an hour or two hours that I can squeeze [for work] on a Sunday.” (G5P2)
Life transition	Navigating new roles or environments, often involving adjusting to changes such as physical separation from loved ones.	“I’m used to doing the weekend with friends or my partner, and if I do not meet with them, then I feel a bit sad.” (G4P3)
Job instability	Perceived uncertainty or insecurity in one’s employment, such as being in a new role, on probation, or unclear about career direction.	“There’s a bit of uncertainty about where I’m going to end up, and that’s always in the back of my mind.” (G3P5)
High ambition	A strong internal drive for career advancement or visibility, often accompanied by self-imposed stress or performance pressure.	“You want to have a promotion within the inside. You want to be visible. And that creates a bit of pressure you put on yourself as well.” (G3P5)
Workplace social comparison	Comparing oneself to colleagues or peers, often resulting in feelings of inadequacy or underachievement.	“I get a lot of pressure from others because [I feel] they did very well, but I did not.” (G5P3)
Personal vulnerability	Being in a mentally or physically vulnerable state, such as experiencing low motivation, burnout, or existential doubts.	“The past few weeks I felt a bit burned out, so at Sunday night, I was filled with a bunch of negative feelings.” (G6P3)

#### 5.2.3 Mitigators

Participants mentioned several factors that helped reduce the intensity or frequency of their “Sunday Blues” (see Table 9). These included both contextual and social elements. A pleasant physical workspace, coupled with the presence of supportive colleagues, was frequently cited as making the return to work feel less daunting. Certain work-related conditions also served to ease the transition. For instance, periods of lighter workload allowed participants to re-engage with their responsibilities more gradually, while autonomy over their schedule, pace, or work location provided a greater sense of control. Beyond these external factors, participants highlighted the role of intrinsic motivation—such as genuine interest in their projects or a broader sense of career satisfaction—in helping reframe work not as a burden, but as a meaningful and purposeful pursuit.

**Table 9:** Mitigators for the “Sunday Blues.”

Code	Explanation	Example participant statement(s)
Pleasant workspaces	A physically comfortable and esthetically pleasing environment that makes returning to work feel more inviting.	“I’m very glad with my workspace because I’m sitting next to a very big window from floor to ceiling … you get to see the daylight all day.” (G2P3)
Healthy work relationships	Positive interpersonal dynamics, such as friendly colleagues or understanding managers, which foster emotional security.	“I have a pretty relaxed life right now because I do not have a lot of people around me to push me.” (G2P2)
Slow work periods	Periods of reduced workloads or less demanding schedules that allow for a more gradual transition back into work.	“Recently, because the summer is coming, my workload is a little bit lower, so I feel okay.” (G6P2)
Flexibility and autonomy	The ability to manage one’s own schedule or work location that diminishes rigid return-to-work stress.	“With the PhD, I do not feel that much actually, because it feels that your work schedule is so flexible as compared to being in a job … because I can swap days or work times.” (G5P2)
Enthusiasm for ongoing work	Genuine interest or excitement about current tasks that makes work a source of purpose rather than a burden.	“I do not feel I have too much ‘Sunday Blues,’ because many of my work I feel internally motivated to do so.” (G5P1)
Career satisfaction	A deep sense of contentment with one’s overall career path or professional identity.	“I also want to be back to work because I love my job … I will not miss it for the world.” (G2P2)

### 5.3 Coping strategies for the “Sunday Blues”

How do employees cope with the “Sunday Blues”? Our analysis uncovered three thematic clusters, including relief-focused, balance-focused, and resilience-focused coping strategies. Notably, many strategies aligned with our *a priori* framework—Desmet’s (2015) categorization of mood-regulating activities, while context-specific strategies such as “plan the weekend thoughtfully” or “prepare for the coming week” also emerged as key approaches.

#### 5.3.1 Relief-focused coping strategies

Participants described a wide range of strategies focused on alleviating the negativity or intensity of the “Sunday Blues” (see Table 10). Many reported deliberately distracting themselves through hobbies or social interactions, while others engaged in activities chosen for their mood-boosting qualities, ranging from relaxation and comfort to enjoyment and rejuvenation. Self-reward was a common approach, often taking the form of indulgences or self-granted privileges. Some participants found relief by openly expressing their feelings, either to process their thoughts or to receive supportive feedback, whereas others preferred to suppress their feelings and maintain a sense of normality to avoid amplifying their distress. Efforts to adopt more positive perspectives were also prevalent, with participants imagining ideal future scenarios or recalling past successes and joyful moments. Additionally, several individuals described drawing on personal traits, such as confidence, to foster a sense of control, or humor, to make the situation feel less difficult.

**Table 10:** Relief-focused coping strategies for the “Sunday Blues.”

Code	Explanation	Example participant statement(s)
Seek distraction	Shifting attention away from distress by engaging in hobbies, chores, entertainment, or social interaction.	“You have something else to think about because you are talking with someone else.” (G3P3)
Seek relaxation	Calming the body and mind through restful practices, such as meditation, deep breathing, or quiet solitude.	“I might shut myself down sometimes … kind of lie on my bed … do some meditation.” (G2P3)
Seek comfort	Turning to people, pets, or familiar objects to feel safe or at ease, such as drinking a favorite beverage, cuddling with pets, or seeking care from loved ones.	“If I stay with my boyfriend and I feel my ‘Sunday Blues’ at that moment, I would like to ask for some comfort, like a hug or support from him.” (G6P4)
Seek joy	Engaging in activities that bring laughter and amusement, such as watching comedies or playfully chatting with friends.	“I like sending memes [to my friends] because it makes me laugh and lightens up the situation.” (G4P3)
Seek refreshment	Taking actions that leave one feeling mentally and physically rejuvenated, like going for a walk outdoors or taking a long shower.	“Long shower. That’s actually better … It’s like you have a new version of yourself, and it feels like stronger and more powerful than before.” (G6P4)
Reward oneself	Giving oneself small treats or pleasures, such as indulging in a TV series, snacks, or window shopping.	“I do a lot of window shopping. It’s like a gift that can bring you some motivation to work again.” (G6P1)
Vent the feelings	Expressing feelings openly, either by speaking to others or writing in a journal.	“If I write my feelings down, then they are not that bad anymore.” (G3P2)
Repress the feelings	Suppressing or ignoring difficult feelings by denying them or acting as if everything is normal.	“If I do not keep thinking about it, it’s just a ‘fake’ feeling.” (G5P4)
Think positively	Focusing on the bright side of situations, such as the remaining time to relax, past achievements, or hopeful future outcomes.	“What you think was, ‘Oh, but I’m only going to work on it tomorrow. I do not have to do it now.’” (G2P2)
Recall happy memories	Looking back on meaningful or happy past experiences, often by revisiting photo albums or social media posts.	“It’s like the reminiscence therapy … making you feel that your life was not wasted … you had a lot of beautiful memories.” (G6P2)
Use personal traits	Relying on one’s inherent strengths, such as resilience, humor, or a confident mindset to manage emotional discomfort.	“Even if these thoughts do creep in, I also know that it’s not going to stick for long, and I believe it does not affect me as much as I think.” (G6P5)

#### 5.3.2 Balance-focused coping strategies

Participants reported various strategies to address the causes of the “Sunday Blues,” with a shared emphasis on balancing personal resources and external demands (see Table 11). A common approach involved enhancing overall health, with many highlighting the importance of rest or exercise to build the energy needed for the week ahead. To reduce external pressures, participants described actions such as avoiding Monday meetings, silencing work notifications over the weekend, postponing non-urgent tasks, or declining social commitments. Another frequently used strategy was structuring the weekend to balance productivity and relaxation; many planned activities that felt enriching or fulfilling, while several extended the weekend experience by staying up late. Others, by contrast, preferred to mentally prepare for the week by organizing tasks or scheduling post-work activities like social outings to create a sense of anticipation. A subset found that engaging in work helped reduce anxiety about unfinished responsibilities, whereas others built clear boundaries between work and leisure, distancing themselves from work-related devices and conversations to preserve the weekend’s restorative potential.

**Table 11:** Balance-focused coping strategies for the “Sunday Blues.”

Code	Explanation	Example participant statement(s)
Improve overall health	Enhance physical and mental health through restorative actions, such as rest or energizing exercises.	“I’ve preemptively been trying to go running more on Sundays because then I feel better in general.” (G6P5)
Avoid work-related stressors	Steer clear of triggers like Monday meetings, deadlines, or work notifications during the weekend.	“Better to not have a deadline [on] Monday, because I do not like spending the last time on working.” (G4P4)
Reduce upcoming responsibilities	Minimize non-essential obligations by postponing certain tasks or declining social invitations that feel draining.	“I feel better to cut things out because you realize these different aspects contributing to your overall stress.” (G2P2)
Plan the weekend thoughtfully	Deliberately structure weekend time by balancing productivity and leisure, and incorporating enriching or relaxing activities.	“Plan well ahead of Sunday to be realistic about how much time I have on weekends, and kind of priorities fun things but also productive things in a good way.” (G4P1)
Extend the weekend	Prolong the weekend experience by delaying regular routines, such as staying up late on Sunday or starting work later on Monday.	“Sometimes I even just go to sleep at 2:00 a.m. on Sunday night. Just to extend the weekend.” (G6P2)
Prepare for the coming week	Get mentally ready for the week ahead by organizing upcoming tasks, choosing outfits, or preparing meals.	“Those things are intertwined as a ‘huge block.’ That’s why it feels so terrifying. But if you break them down, it’s just small tasks.” (G5P4)
Create positive expectations	Plan things to look forward to, such as hobbies, post-work social events, or upcoming holidays.	“I also plan something different from work, like workouts, or a dinner with friends after work.” (G4P4)
Work	Engage in limited work to complete pending tasks or maintain a productive rhythm.	“Once something is done, I’m suddenly more optimistic.” (G2P2)
Maintain clear work-life boundaries	Keep firm boundaries between work and leisure time during weekends by disconnecting from work devices or communications.	“I started my work three months ago, and up to now, I’ve only brought [my laptop] once with me on one weekend, and I want to keep it that way.” (G3P3)

#### 5.3.3 Resilience-focused coping strategies

Participants mentioned four strategies centered on fostering long-term emotional resilience and wellbeing (see Table 12). One common approach was deep reflection and analysis, with participants seeking to understand the patterns and root causes behind their mood state, which often led to greater clarity, potential solutions, or strategies for prevention. In contrast, others chose to simply allow the “Sunday Blues” to unfold, accepting these negative feelings as a natural and transient part of life rather than something to be resolved. Some went further by reframing their experience in a constructive light, viewing the “Sunday Blues” as a catalyst for productivity, a means to connect with others, or an opportunity for personal growth. Additionally, several participants shared coping skills they had acquired through therapy or past experiences, underscoring the importance of self-regulation and the long-term value of building mood resilience.

**Table 12:** Resilience-focused coping strategies for the “Sunday Blues.”

Code	Explanation	Example participant statement(s)
Analyze patterns and causes	Engage in reflective thinking to understand the “Sunday Blues,” including its patterns, underlying causes, and potential solutions.	“I try to understand why I have it, and what is the cause. And in that way, maybe next time I can do better to get rid of it.” (G4P2) “When I filled in the diary, it was amazing that I understood what’s happening … so, it wasn’t about Monday, it was about a struggle with my son.” (G1P2)
Embrace the feelings	Accept the “Sunday Blues” as a natural part of weekly emotional rhythms, allowing the feeling without trying to suppress, fix, or avoid it.	“Sometimes you have to just let it happen. You should allow yourself to feel sad at some moment.” (G4P4) “Actually, the negative part is normal, and if you take it as a normal thing, it will not harm you so much.” (G6P4)
Reframe the experience	Reinterpret the “Sunday Blues” in a constructive way, viewing the experience as potentially motivating, meaningful, or a tool for personal growth or social connection.	“I would rationalize it and then place it in a way like, ‘I’m stressed because I’m nervous and I want to do well. Maybe the stress is going to help me perform better.’” (G6P5) “I use it as one of the very small tricks to build connection with my partner. I know she probably has more severe ‘Sunday Blues’ compared to mine. So, it’s a way to start a conversation and provide a chance for her to vent the emotions.” (G5P1)
Develop self-regulation skills	Develop emotional self-regulation skills through personal practice or therapeutic support to remain calm, centered, and proactive in everyday mood management.	“I had some hypnotherapy and learned some tricks for breathing and relaxing. So when it was stressful, or I felt very down, I learned how to cope. That helped me very much.” (G2P3)

## 6 Discussion

This study examined how employees experience and cope with the “Sunday Blues.” The findings provide a more detailed account of this culturally familiar phenomenon by revealing its varied manifestations, the conditions surrounding its occurrence and intensity, and the different ways employees respond to it. We first synthesize the principal findings, then discuss their theoretical and practical implications and the study’s limitations.

### 6.1 Principal findings

First, participants’ accounts showed that the “Sunday Blues” involves multiple components. Affectively, participants described loss-oriented feelings, such as sadness, regret, and emptiness, as well as threat-oriented feelings, such as anxiety and stress. These feelings appeared to reflect two intertwined temporal orientations: looking back at the closure or perceived incompleteness of the weekend and looking ahead to the demands of the coming workweek. The experience could therefore combine retrospective distress concerning whether the weekend had provided sufficient rest, fulfillment, or personal time with anticipatory stress or anxiety concerning the approaching workweek. Participants’ accounts also showed that these feelings were accompanied by bodily discomfort and arousal, cognitive patterns such as rumination and self-criticism, and behavioral tendencies such as procrastination, withdrawal, and disrupted sleep. Thus, despite the “blues” in its name, sadness captured only part of the affective experience, while affect itself represented only one component of the broader phenomenon.

Second, the findings suggest that “Sunday Blues” is shaped by both immediate Sunday-evening triggers and broader work-life conditions. Immediate triggers included negative evaluations of how the weekend had gone and moments when upcoming work demands became psychologically present. Its intensity, however, appeared to depend partly on broader conditions. Blurred work-life boundaries, job instability, and personal ill-being could increase susceptibility to more intense experiences, whereas autonomy, supportive work relationships, and job satisfaction could make the coming week feel more manageable or worthwhile and thereby mitigate the experience. Thus, despite the temporal emphasis on “Sunday” in the term, the “Sunday Blues” cannot be attributed simply to the weekend ending or Monday approaching. It also reflects how the weekend has unfolded, what the upcoming workweek is expected to hold, and the personal resources and contextual conditions surrounding that transition.

Third, participants’ coping strategies showed that managing the “Sunday Blues” involves more than reducing unpleasant feelings on Sunday evening. Relief-focused strategies, including distraction, self-reward, and positive thinking, were used to soften the immediate intensity of the experience. Balance-focused strategies, such as planning the weekend, reducing upcoming pressures, and maintaining boundaries between work and leisure, sought to rebalance personal resources and external demands. Resilience-focused strategies, including reflection, acceptance, and reframing, approached the “Sunday Blues” as a recurring pattern to be understood, accepted, or transformed over time. Coping can therefore be understood as regulation of the broader transition between personal time and work time, operating across different time horizons.

**Figure 1:**
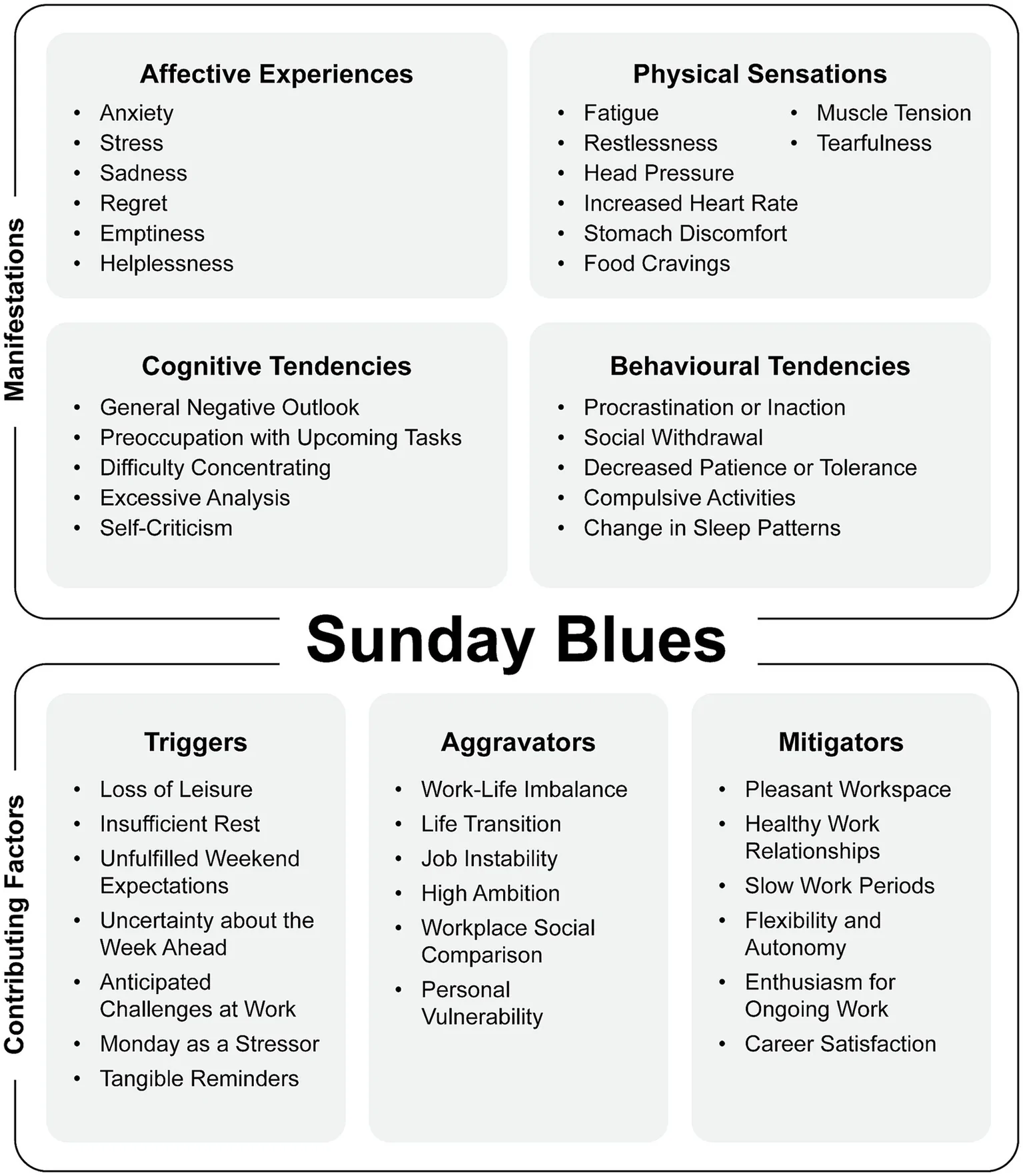
An integrative framework for understanding the “Sunday Blues”.

Figure 1 synthesizes these findings in an integrative framework that consists of the core components and contributing factors of the “Sunday Blues.” Rather than constituting a formal causal theory, the framework organizes the phenomenon as a multidimensional weekend-to-workweek transition experience activated by immediate triggers and shaped by broader work-life conditions. Coping strategies are presented separately because they represent responses to the experience rather than its manifestations or antecedents. Together, the framework and coping typology provide complementary accounts of what the “Sunday Blues” is, what shapes it, and how employees respond to it.

### 6.2 Theoretical implications

This study makes three theoretical contributions. First, the findings clarify the “Sunday Blues” as a multidimensional experience. Existing research has described it variously as unease, dread, anxiety, or depression-like feelings (e.g., Mihalcea and Liu, 2006; Tufvesson, 2022; Zuzanek, 2014). Our findings suggest that these descriptions do not necessarily represent competing understandings. Instead, they may capture different affective components within a broader configuration of physical, cognitive, and behavioral manifestations. Conceptualizing the “Sunday Blues” as an umbrella term therefore cautions against reducing it to a unitary mood state and underscores the need for future research to specify which dimensions or manifestations are being examined or measured.

Second, the findings broaden the temporal understanding of the “Sunday Blues.” Existing research on weekly rhythms locates changes in mood within the progression of the weekly calendar (e.g., Akay and Martinsson, 2009; Zuzanek and Mannell, 1993). Recovery research emphasizes how experiences during the weekend carry into its closing period (e.g., Fritz et al., 2010; Fritz and Sonnentag, 2005), whereas work anticipation research focuses on how future work demands become psychologically present before work resumes (e.g., Casper and Sonnentag, 2020). Our findings add to these perspectives by suggesting that present experiences of the “Sunday Blues” can involve both retrospective appraisals of the weekend and prospective appraisals of the approaching workweek. This temporal distinction cautions against interpreting the phenomenon solely as either an anticipatory response to Monday or a consequence of insufficient weekend recovery. Instead, the two temporal orientations may coexist and jointly constitute the experience.

Third, the findings provide an initial conceptual foundation for further theory development and empirical research on the “Sunday Blues.” The integrative framework organizes the phenomenon’s manifestations and contributing factors and can be further developed, refined, and empirically examined. Future quantitative studies could investigate the prevalence, intensity, and variability of the “Sunday Blues” across occupational groups, work arrangements, cultures, and life situations. They could also explore questions prompted by the qualitative findings, such as whether unfulfilled weekend expectations are associated with regret or sadness, whether anticipated work demands are associated with anxiety, rumination, or sleep disruption, and whether autonomy or job satisfaction is associated with less intense experiences. Longitudinal or experience-sampling designs would also be valuable for examining how immediate Sunday-evening triggers and broader work-life conditions jointly shape the onset and development of the “Sunday Blues” over time. In addition, intervention-oriented studies could examine whether different coping strategies operate through different mechanisms, such as short-term emotional relief, restoration of perceived control, or improved boundary management.

### 6.3 Practical implications

The findings offer practical value without implying that the “Sunday Blues” is inherently a clinical condition. For employees, the framework can provide language for recognizing whether their experience is primarily related to an unfulfilling weekend, anticipated work demands, or broader work-life conditions. The catalog of 24 coping strategies can then support reflection on their current responses and exploration of potential alternatives. In clinical contexts, the framework may also help practitioners explore whether recurrent experiences of the “Sunday Blues” form part of a broader pattern of anxiety, depression, occupational strain, or work-life conflict, while avoiding treating an everyday temporal transition as a diagnosis in itself.

The findings can also inform the development of mood-regulation interventions aimed at alleviating or managing the “Sunday Blues.” Researchers and practitioners in mental healthcare, organizational management, and design research may draw on our findings to inspire innovation. Here, we propose six potential directions for such efforts. Noteworthily, these directions are grounded in the qualitative findings but remain to be developed and empirically evaluated.

#### 6.3.1 Easing weekend-to-workweek transition

The contrast between weekend leisure and weekday obligations can make the transition emotionally challenging. Interventions such as guided journaling or reflective board games could support the development of end-of-week rituals, helping individuals reflect on their weekend experiences and identify realistic and manageable intentions for the coming week.

#### 6.3.2 Enhancing weekend fulfillment

When weekend experiences fall short of expectations, individuals may feel regret or disappointment. Digital or physical planning tools could help individuals structure their weekends to balance necessary tasks with relaxation and meaningful engagement, without turning leisure into another source of performance pressure.

#### 6.3.3 Promoting mental readiness for Monday

Anticipatory stress about Monday’s responsibilities often emerges on Sunday evening. Interventions could support mental preparation through activities like expressive writing or socially supportive interactions, helping individuals process their thoughts and identify manageable first steps for the week ahead.

#### 6.3.4 Reducing tangible work-related stressors

Organizational policies that discourage unnecessary work-related communication during weekends could limit the extension of work stress into personal time. In parallel, personal digital assistants could automatically mute work notifications or schedule device downtime to support psychological detachment from work.

#### 6.3.5 Leveraging environmental or organizational mitigators

A welcoming work environment can ease the return to work on Monday. Environmental improvements, such as natural lighting or indoor plants, could be explored as ways of making the workplace more welcoming. Institutions might also offer flexible arrangements, such as personalized “weekend” days or delayed Monday start times where feasible, to support smoother re-entry into the workweek.

#### 6.3.6 Building emotional resilience

The findings suggest that personal resilience may support the management of recurring mood fluctuations. Organizations could integrate resilience-building programs, such as workshops on emotional self-regulation, into professional development offerings. Individuals may also benefit from self-guided toolkits, such as coping strategy cards or serious games, designed to help them learn and reinforce coping skills for everyday use.

### 6.4 Limitations of the study

This study has several limitations. First, all participants worked in the Netherlands, and their lived experiences were necessarily situated within Dutch workplace norms and societal expectations. Recruitment through a university-based research pool and the researchers’ professional networks may also have limited the diversity of the sample. In particular, a substantial proportion of participants worked in higher education, meaning that the findings may partly reflect the particular rhythms, pressures, and work-life boundaries of academia. The sample nevertheless included participants from healthcare, transportation and logistics, food and beverage, elementary education, professional services, semi-government, wholesale and retail sales, and information technology and services. Future research should examine the transferability of the findings across more varied cultural and occupational settings.

The eligibility criterion required participants to frequently experience, or to have previously experienced, low moods on Sundays in anticipation of the workweek. Although this criterion enabled detailed and information-rich accounts, it may have favored more recurrent, intense, or negatively perceived forms of the “Sunday Blues.” The findings should therefore not be interpreted as evidence about the prevalence or typical intensity of Sunday-evening mood experiences among employees more broadly, including those who rarely experience such feelings. Moreover, because the study centered on low Sunday moods, it was not designed to capture the full range of positive and negative affect that employees may experience during the weekend-to-workweek transition. Future research could include employees with varying levels of familiarity with the phenomenon to examine how the “Sunday Blues” differs across individuals and contexts, whether positive and negative feelings coexist during this period, and how such emotional ambivalence may shape the experience or its management.

Finally, although all focus groups followed the same procedure, their demographic composition varied. Some groups were relatively homogeneous in age and occupational background, whereas others included a wider range of career stages and working domains. These differences may have influenced discussion dynamics, including participants’ willingness to share, their sense of mutual identification, and the issues that became prominent. Future research could use purposive sampling or quotas based on age, occupation, career stage, and work arrangement to support more systematic comparisons across subgroups.

## 7 Conclusion

This study clarifies the “Sunday Blues” as more than a dip in mood at the end of the weekend. It is a multidimensional weekend-to-workweek transition experience involving affective, physical, cognitive, and behavioral manifestations. The experience may also involve two temporal orientations: retrospective distress concerning the weekend’s ending or perceived incompleteness and anticipatory stress or anxiety concerning the approaching workweek. Immediate triggers activate the experience, while broader personal and work-life conditions can intensify or alleviate it. Employees respond through strategies directed toward immediate relief, the rebalancing of demands and resources, or longer-term resilience. By clarifying its manifestations, contributing factors, and coping strategies, the study enhances understanding of a culturally familiar but scientifically underexplored phenomenon and provides a foundation for future empirical research and intervention development.

## Data Availability

The datasets generated and analyzed during the current study are not publicly available because participants did not provide written consent for their full data to be shared. Due to ethical and privacy considerations, the underlying qualitative data cannot be made available. However, anonymized participant quotes are included in the manuscript to illustrate the generated codes and themes and to support transparency in the reporting of the analysis. Requests to access the datasets should be directed to the corresponding author.
