# Supplementary material for: The “Sunday Blues”: a qualitative exploration of manifestations, contributing factors, and coping strategies among employees
Source: Front Psychol. 2026 Sep 4;17:1882556. doi: 10.3389/fpsyg.2026.1882556 (PMC13585627; doi:10.3389/fpsyg.2026.1882556)
Supplement: Supplementary file 1 [file Supplementary_file_1.docx]

Supplementary materials

# The supplementary materials include:

1. Sensitisation materials: Online Sunday night diary (p. 2)

2. Focus group guide (p. 7)

3. Prompting materials: Factor cards and strategy cards (p. 10)

4. Self-care booklet (p. 12)

1. Sensitisation materials: Online Sunday night diary

Table 1. Instructions and questions from the online Sunday night diary.

| Section | Instruction/Question |
| --- | --- |
| Onboarding | Welcome to your Sunday reflection space! We’ll guide you through a few prompts to help you reflect on your mood, review your weekend, and look toward your week ahead. Grab your favorite drink, find a cozy spot, and let’s gently explore some thoughts together. |
| Weekend review | Let’s get started by journaling about your weekend. |
|  | In general, my weekend was...  What did you do? Who were you with? |
|  | A highlight of my weekend was...  What makes it a highlight? What has it brought you? |
|  | A lowlight of my weekend was...  What makes it a lowlight? How did it influence you? |
| Mood shifts | Let’s take a closer look at how your Sunday has been. |
|  | Overall, during the daytime on Sunday, I was feeling...  Choose one or more character(s) that best describe(s) your mood during the daytime on Sunday.  Please express in your own words what feeling(s) the character(s) represent(s).  Please reflect on and explain why you felt this way. |
|  | At this moment right now, I am feeling...  Choose one or more character(s) that best describe(s) your current mood.  Please express in your own words what feeling(s) the character(s) represent(s).  Please reflect on and explain why you feel this way. |
| Week ahead | Now, let’s explore what the upcoming week looks like for you. |
|  | Things I look forward to in the upcoming week are...  Are there any specific reasons that you are looking forward to them? |
|  | Things I do not look forward to in the upcoming week are...  Are there any specific reasons that you are not looking forward to them? |
| Mood awareness and coping | Great job, you’re almost done with today’s journaling—only three questions to go! Journaling provides a chance to reflect and have a deep conversation with yourself. Through this, you're getting to know yourself a little more. Now, let’s finish up by checking in with yourself. |
|  | What insights did you gain from today’s journaling, and what would you like to remember from this session? |
|  | Are you generally aware of your mood?  If yes, what clues help you? If not, can you reflect on what might be the reason? |
|  | If you experience unpleasant feelings on Sunday evening or night, do you have ways to cope with them?  If yes, can you reflect on how you cope with the unpleasant feelings? Do you have particular strategies? |
| Closing | Thank you for taking the time to reflect with us! You’ve done a great job today. We hope your upcoming week is filled with joy, purpose, and balance. Remember, you’ve got this. |

Figure 1. Example interfaces of the online Sunday night diary on the Qualtrics platform.

Figure 2. Template summarising participants’ two Sunday night diaries, used as a final sensitisation step.

2. Focus group guide

Table 2. Focus group guide.

| Stage | Focus | Instruction/Question |
| --- | --- | --- |
| Warm-up | Basic information | Please pick one or more meme cards that best represent how you’re feeling right now, and tell us who you are, what you do, and why you chose the card(s). |
|  | Ethical considerations | This session will be recorded on video and audio, but any personal information will be kept anonymous. If you ever feel uncomfortable with a topic, please let us know—you can skip any question or stop participating at any time. |
|  | Ground rules | Please try to talk to each other as much as possible, rather than only responding to the researchers. We’re interested in your real-life experiences, not scientific explanations, so there are no right or wrong answers—feel free to share openly. |
| Exploring the Sunday Blues | Working definition | As you may know, the “Sunday Blues” is the not-so-great feeling we get when the weekend ends and the new workweek is just around the corner. |
|  | General experience | Can you describe a recent time when you felt the Sunday Blues? |
|  |  | How often do you experience the Sunday Blues? |
|  |  | When do you usually start to notice the Sunday Blues kicking in? |
|  | Micro experiences | What specific feelings do you have when you experience the Sunday Blues? |
|  |  | When you have the Sunday Blues, do you notice any symptoms in your body? |
|  |  | How do you view your life or the world around you when you’re experiencing the Sunday Blues? |
|  |  | If someone interacts with you when you’re feeling the Sunday Blues, how do you usually respond to them? |
|  |  | What kind of behaviors or actions do you notice in yourself when you have the Sunday Blues? |
|  |  | When you’re feeling the Sunday Blues, what activities do you feel like doing, and what do you prefer to avoid? |
| Understanding influencing factors | Personal experiences | What specifically makes you feel the Sunday Blues?  Is it something about your work, your personal life, or something else?  Why do you think this particular thing causes your Sunday Blues? |
|  | Supplementary insights | Is there any factor on the “factor cards” that you can relate to, besides what we’ve already discussed?  Why do you think this factor affects you? |
| Identifying coping strategies | Personal experiences | When experiencing the Sunday Blues, what do you usually do to make you feel better?  Do you talk to someone, do something fun, or have any other methods?  How does this help you? Why do you think it helps (or doesn’t)? |
|  | Supplementary insights | Is there any strategy or activity on the “strategy cards” that you’ve tried before, besides what we’ve already discussed?  How did you use this strategy or did this activity?  How does it work for you? Why do you think it’s effective (or not)? |
| Wrap-up | Extra information | Please choose one or more meme cards that capture how you’d ideally like to feel on a Sunday night, then share the card(s) you picked, the mood you’re aiming for, and why. |

3. Prompting materials: Factor cards and strategy cards

Figure 3. “Factor cards” used to stimulate further reflections on contributing factors to the “Sunday Blues.”

Figure 4. “Strategy cards” used to stimulate further reflections on coping strategies for the “Sunday Blues.”

4. Self-care booklet

Figure 5. Self-care booklet provided to participants for managing the “Sunday Blues” after the focus group.
